# OXS2 is Required for Salt Tolerance Mainly through Associating with Salt Inducible Genes, *CA1* and *Araport11*, in *Arabidopsis*

**DOI:** 10.1038/s41598-019-56456-1

**Published:** 2019-12-30

**Authors:** Ying Jing, Lin Shi, Xin Li, Han Zheng, Jianwei Gao, Mei Wang, Lilong He, Wei Zhang

**Affiliations:** 1 Institute of Vegetables and Flowers, Shandong Key Laboratory of Greenhouse Vegetable Biology, Shandong Branch of National Vegetable Improvement Center, Huanghuai Region Vegetable Scientific Station of Ministry of Agriculture (Shandong), Shandong Academy of Agricultural Sciences, Jinan, 250100 China; 2 Key Laboratory of Plant Development and Environment Adaptation Biology, Ministry of Education, School of Life Science, Shandong University, Qingdao, 266237 China

**Keywords:** Plant cell biology, Plant molecular biology, Plant physiology, Plant stress responses

## Abstract

Salt stress is one of the abiotic stresses affecting crop growth and yield. The functional screening and mechanism investigation of the genes in response to salt stress are essential for the development of salt-tolerant crops. Here, we found that *OXIDATIVE STRESS 2* (*OXS2*) was a salinity-induced gene, and the mutant o*xs2-1* was hypersensitive to salt stress during seed germination and root elongation processes. In the absence of stress, OXS2 was predominantly localized in the cytoplasm; when the plants were treated with salt, OXS2 entered the nuclear. Further RNA-seq analysis and qPCR identification showed that, in the presence of salt stress, a large number of differentially expressed genes (DEGs) were activated, which contain BOXS2 motifs previously identified as the binding element for AtOXS2. Further ChIP analysis revealed that, under salt stress, OXS2 associated with *CA1* and *Araport11* directly through binding the BOXS2 containing fragments in the promoter regions. In conclusion, our results indicate that OXS2 is required for salt tolerance in *Arabidopsis* mainly through associating with the downstream *CA1* and *Araport11* directly.

## Introduction

Salt stress, which is one of the representative abiotic stresses, reduces plant growth and crops yield worldwide^1,2^. As the overexploitation of farmland, salinization of soil is severely deteriorated. It has been reported that more than 30% of the farmland has been affected by salinity. By the year 2050, over half of farmland will be vulnerable to salt stress^3^. Salt stress increases soil osmotic potential, causes an imbalance of ions, disrupts physiological processes, inhibits plant growth and even leads to plant death^4–10^. Soil with a high concentration of salt reduces water absorption or even causes water extravasation from plant tissues, which leads to physiological drought, plasmolysis, and cell death^10–13^. High Na^+^ and Cl^−^ concentrations decrease enzyme activities in cells and thus restrict plant growth^13–15^. In addition, excess Na^+^ and Cl^−^ can lead to the accumulation of amino acids in plants and cause cell damage and death^16^. Under salt stress, the intracellular reactive oxygen species (ROS) are increased, which damages the plant cell plasma membrane, resulting in the loss of intracellular ions and organic matters^17^. ROS can also cause interference when external toxic ions penetrate into plant cells and inhibit plant growth and development^16,18^. Salt stress leads to a decreased photosynthesis in plants^13^. Low-concentration of salt promotes plant respiration, high-concentration of salt inhibits plant respiration, and excessive salt stress hinders plant protein synthesis^19^.

Salt tolerant crops can be achieved through two approaches. First, salt tolerant crops can be generated by normal hybridization breeding of existing salt tolerance genotypes^20,21^. Second, salt tolerance transgenic crops can be engineered through modifying functional salinity response genes^22^. For traditional breeding methods, the selection of stress tolerance traits is complicated and time-consuming, limiting the quality and the generation rate of tolerance varieties. On the contrary, genetic engineering technologies can provide new salt tolerance crops with specific functionalities in a much-shorter-period of time.

OXIDATIVE STRESS 2 (OXS2), which belongs to a family containing five zinc finger (ZF) proteins with a canonical C2-H2 ZF and two C3-H ZFs, is a classical transcription factor^23^. *OXS2* is widely expressed in plants, such as *Arabidopsis*, maize, rice, etc. Previously *Arabidopsis OXS2* and two *OXS2* homolog genes from maize (*ZmOXS2b* and *ZmO2L1*) have been found to play a role in stress escape and Cd stress tolerance^24,25^. In *Arabidopsis*, under high stress, AtOXS2 promotes plant stress escape by directly binding the promoter of *SOC1*, which is a representative floral transient gene^24^. In maize, the *OXS2* homologous, *ZmOXS2b* and *ZmO2L1*, can confer Cd tolerance when heterogeneously expressed in *Arabidopsis* by activating the promoter of *Cd-Inducible Methyltransferase 1* (*CIMT 1*), which is specifically expressed in root and also enhances the Cd resistant ability of *Arabidopsis* when overexpressed alone^25^. All of these three OXS2 members are able to directly recognize segments including the CT-rich BOXS2 motif. Here, we found that AtOXS2 was required for salt tolerance in *Arabidopsis*. RNA-seq analysis has selected multiply candidates which were controlled by AtOXS2 under salt stress. ChIP experiments suggest that AtOXS2 functions mainly through associating with the downstream salt induced and related genes, *CA1* and *Araport11*, which are regulated by the BOXS2-containning promoters. Our findings suggest a new salt regulation mechanism, which can be potentially used for engineering salt tolerance in major crop plants.

## Results and Discussion

### AtOXS2 is inducible by salt in *Arabidopsis*

**Figure 1:**
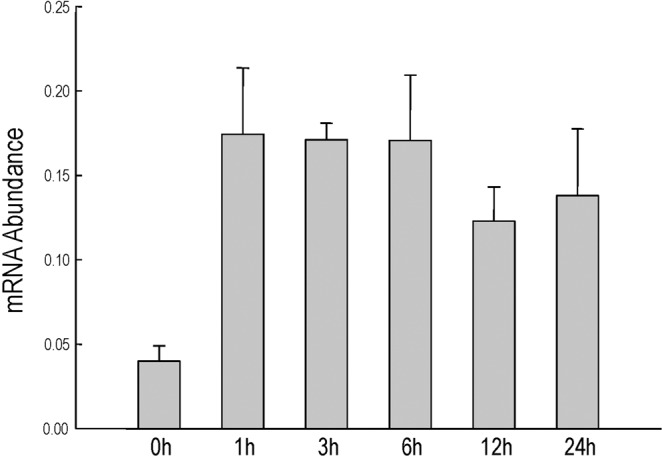
*AtOXS2* transcript abundance in *Arabidopsis* without or with salt stress. *AtOXS2* transcript abundance in *Arabidopsis* seedlings (relative to ACT2 control) determined by RT-qPCR. 10-day-old seedlings were exposed to 150 mM NaCl. Error bars indicate ± SD from three independent experiments.

To test whether the expression of *AtOXS2* is responsive to salt stress, *Arabidopsis* seedlings were grown in ½ MS for 10 d, and then transferred to the hydroponic culture plates with 150 mM NaCl. The whole seedlings were collected at different time points after the salt treatment for quantitative reverse transcript PCR (RT-qPCR). The data indicated that the abundance of the *AtOXS2* mRNA increased within 1 h after the salt treatment and stayed at a high level within the following 24 h (Fig. 1), indicating that the *AtOXS2* transcript is activated in response to salinity stress and may be involved in plant salinity responses. As a classical transcription factor, AtOXS2 may control massive downstream *cis*-elements^24^. Under stress, the upregulation of the mRNA abundance will generate more proteins to activate genes which are related or resistant to the stress.

### AtOXS2 is required for salt tolerance in *Arabidopsis*

**Figure 2:**
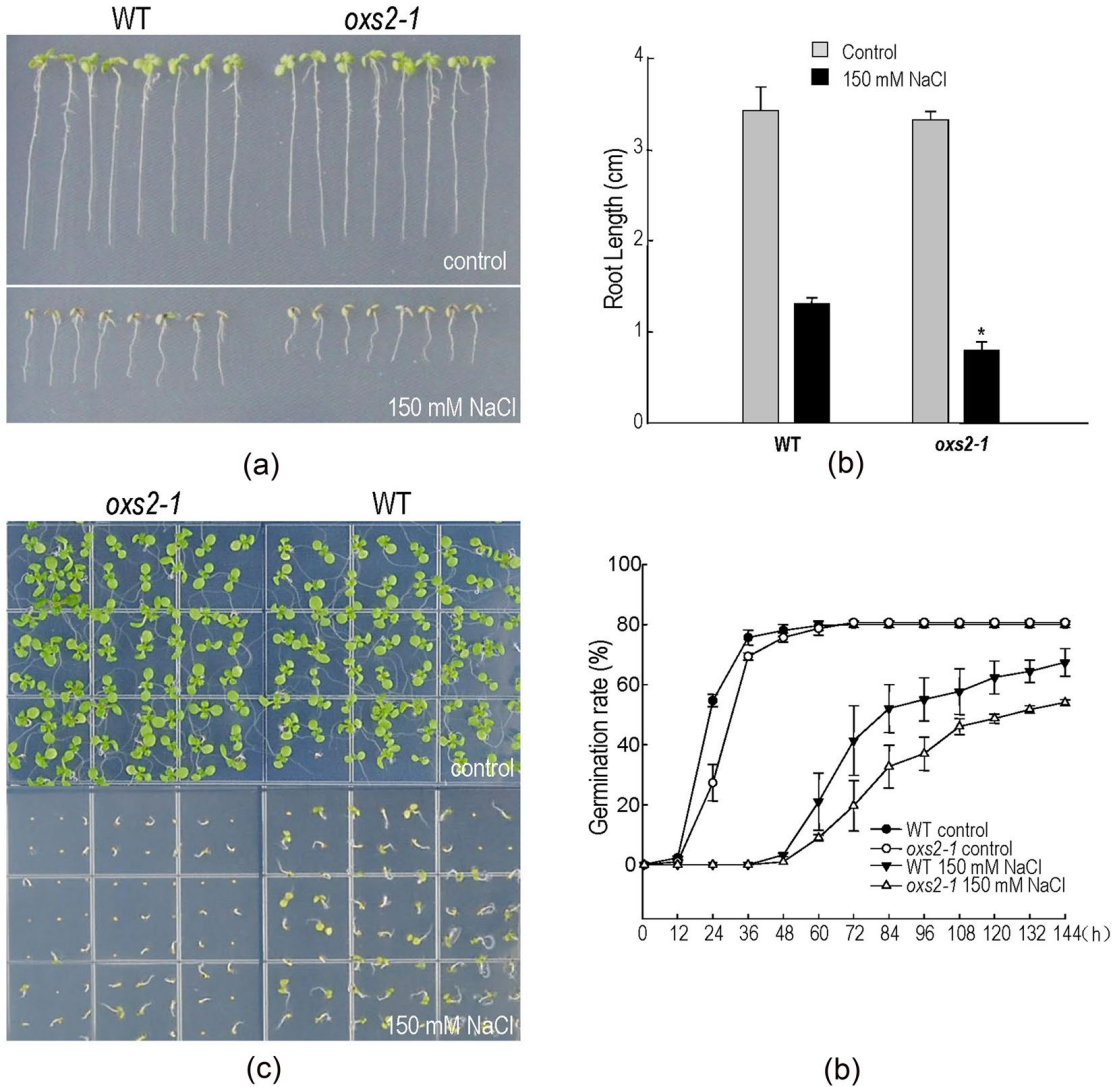
Phenotyping analysis of *oxs2-1* and wild-type plants without or with salt stress. (**a**) *Arabidopsis* plants germinated on ½ MS plates vertically for 3 d were transferred to plates without or with 150 mM NaCl for another 10 d. Representative result from three reproducible experiments was shown. (**b**) Average root length of seedlings cultured as the same growth condition in (**a**). The root length of 5 seedlings of each class was measured as the mean value (remove the top and lowest value). Error bars indicates ± SD from three independent experiments. (**c**) About 60 *Arabidopsis* seedlings were germinated and grown on ½ MS plates horizontally without or with 150 mM NaCl for 10 d. Representative test from three reproducible independent experiments was shown. (**d**) Germination rate of seedlings cultured as the same growth condition in (**c**). Error bars indicate + SD from three independent experiments.

To confirm if AtOXS2 is involved in plant salt tolerance, homozygotes of the T-DNA insertion mutant lacking detectable transcript by RT-PCR were collected for the salt stress test. As shown in Fig. 2(a–d), under the normal condition, the phenotype of the *oxs2-1* plant was indistinguishable from that of the wild-type plants. In salt (150 mM NaCl) supplemented ½ MS plates, the root length of *oxs2-1* was shorter than that of the wild-type plants (Fig. 2(a,b)), and the shoot growth of *oxs2-1* was also poorer than that of the wild-type plants (Fig. 2(c,d)). As germination is a key phenotype for plants to be resistant with salinity, germination rate tests were conducted in the mutants and the wild-type plants. As shown in Fig. 2(d), in the control environment, the germination rate of both *oxs2-1* and wild-type plants had no obvious difference at 60 h. However, in the presence of salinity, the germination rate of *oxs2-1* was lower than that of the wild-type control after 48 h. We also generated more than 5 independent *OXS2*-overexpressing (OE) transgenic lines, and two of these lines were randomly selected for the salt tolerance test, which were identified by RT-qPCR (Supplementary Fig. S1(a)). After three biological tests, we did not see any significant differential phenotype between the wild-type plants and the OE lines (Supplementary Fig. S1(b)). Although the OE lines did not show any significant salt tolerance phenotype, considering *oxs2-1* is significantly salt sensitive, and AtOXS2: FLAG can recover the salt sensitive phenotype of *oxs2-1* (Supplementary Fig. S2), we also conclude that OXS2 is required for salt tolerance in *Arabidopsis*. Considering the *oxs2-1* plant is sensitive against diamide, and overexpressing OXS2 failed to yield plants with higher stress tolerance^24^, it is supposed that there exists a dose-effect for OXS2 to regulate stress tolerance in *Arabidopsis*, and excess OXS2 does not increase the contribution to stress tolerance. In a word, comparing with the OE lines, the salt sensitive phenotype of the *OXS2* mutant is more reliable for validating the function of OXS2. These results suggest that AtOXS2 plays a role in salt stress in *Arabidopsis*.

### AtOXS2 is specifically accumulated in the nuclear under salt stress

**Figure 3:**
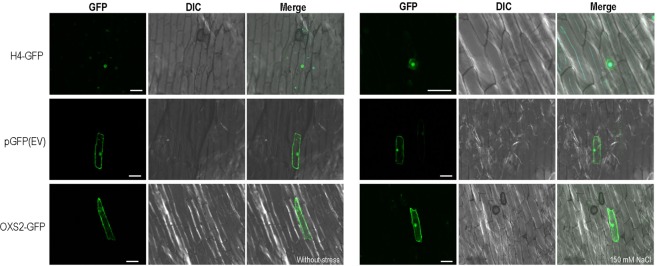
Subcellular localization of H4, OXS2 and empty vector tagged with GFP and transiently expressed in onion epidermal cells on ½ MS for 24 h in the dark at room temperature and transfer to ½ MS with 150 mM NaCl. The images were obtained from the GFP channel, DIC channel, or a merged image of the two channels. Scale bar: 100 μm.

AtOXS2 shows a canonical transcription factor feature and is accumulated in the nucleus under cold or ABA stress. However, there is no evidence supporting the translocation of AtOXS2 into the nuclear under salt stress. To test whether AtOXS2 plays a role as a transcription factor under salt stress, the coding region was fused to GFP expressed transiently in onion epidermal cells. GFP-Histone 4 (H4) specifically expressed in the nucleus was used as a positive control, and the empty vector (pGFP) was used as a negative control. In the absence of salt stress, the AtOXS2 fusion existed in the cytoplasm. However, when treated with 150 mM NaCl, AtOXS2 was translocated into the nucleus, while the location of H4 or GFP was not affected by salt stress (Fig. 3). It is suggested that AtOXS2 specifically entered the nuclear under salt stress. The specific nuclear localization of AtOXS2 could play a role in salt tolerance at the molecular level. These results implied that AtOXS2 might target some downstream *cis*-elements which are required for salt stress responses in *Arabidopsis*.

### Differentially regulated genes identified from RNA-Seq analysis

**Figure 4:**
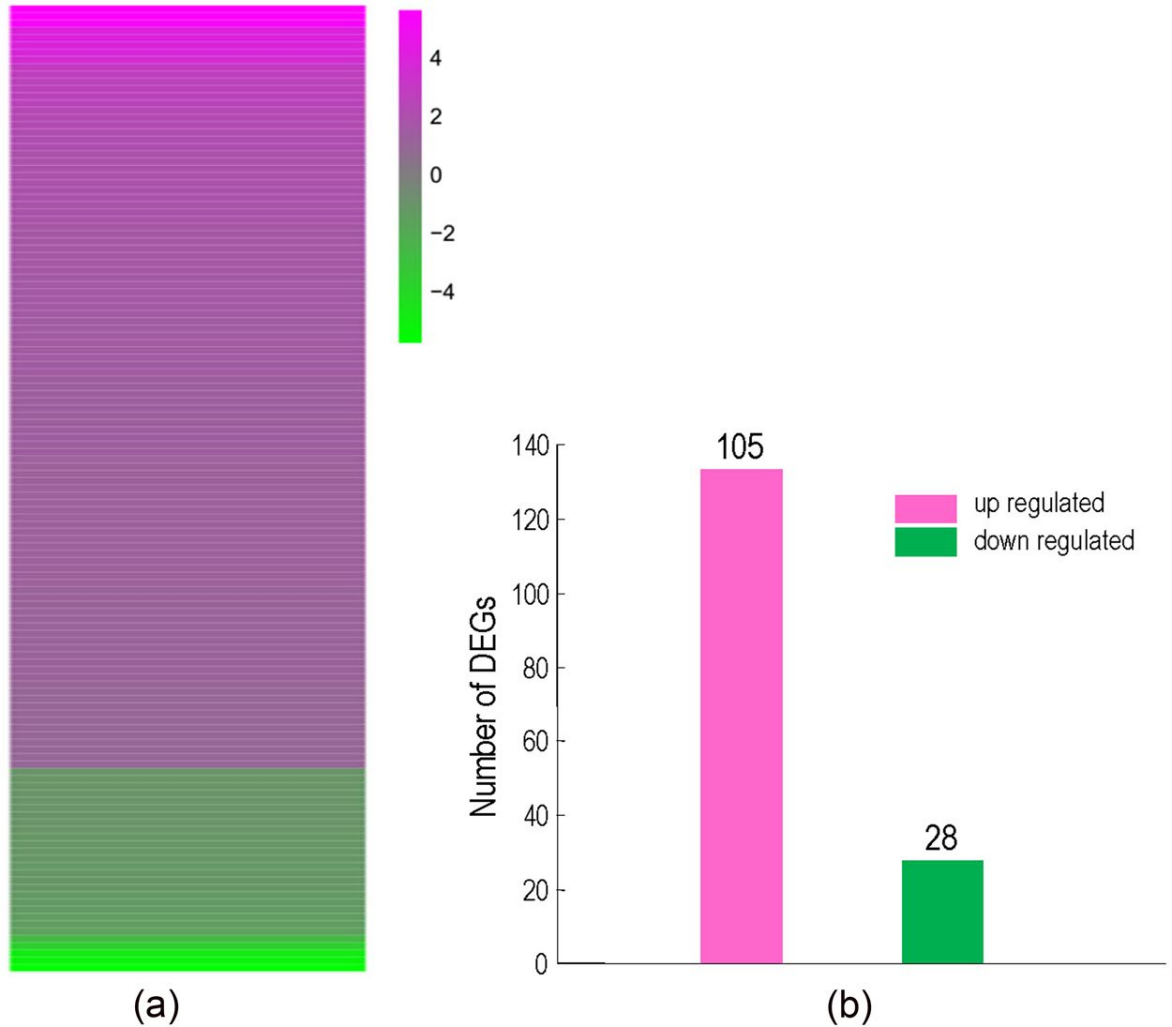
RNA-seq analysis. Heat map of clustering analysis of the 133 DEGs in *oxs2-1* VS WT group. (**a**) Expression ratios shown as log2 values. Magenta represents increased expression; green represents decreased expression compared to *oxs2-1*. Vertical axis shows fold enrichment of relative transcript levels between *oxs2-1* and wild-type plants. (**b**) Numbers of DEGs in in *oxs2-1* VS WT group.

The salt-sensitive phenotype associated with the loss of AtOXS2 is likely due to the deletion of the transcription factor, AtOXS2 may recognize salt tolerance related genes from the protein and DNA interaction. To examine whether the gene expression pattern is affected, an RNA-seq analysis was carried out for the salt-treated *oxs2-1* and wild-type plants. DEGs with statistically significant changes (up-regulated by at least 2-fold or downregulated by at least 0.5-fold, with a corrected P-Value < 0.05) were selected. The total number of DEGs is 133 with 105 up-regulated DEGs and 28 down-regulated DEGs (Fig. 4(a,b)). Loss of AtOXS2 may lead to a decreased expression of the downstream genes. Thus, we narrowed the DEGs into 28 down-regulated DEGs, which were named from DEG1 to DEG 28 according to the RNA-seq ranking (Supplementary Table S1).

### Validation of differential expression

**Figure 5:**
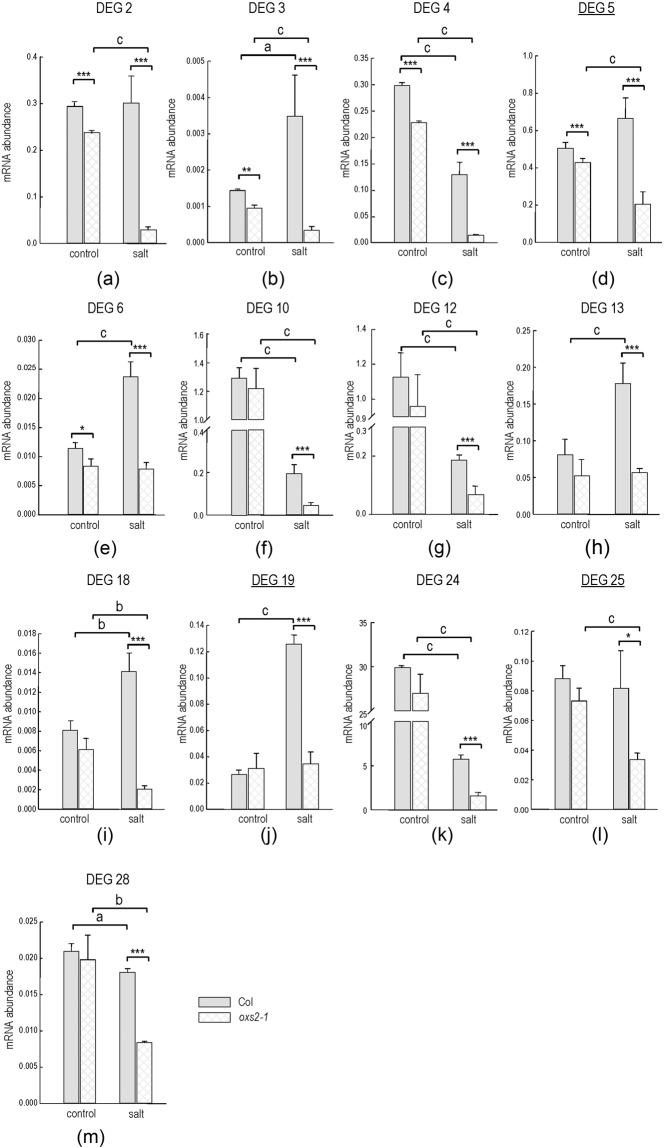
Expression of the selected DEGs (relative to ACT2) (**a**–**m**) in 13-d-old seedlings exposed to 0 or 150 mM NaCl. Error bars indicate ± SD from three independent experiments. P value of Student’s t test: wild-type compared with *oxs2-1*. *P < 0.05; **P < 0.01; ***P < 0.001; Control compared with salt stress. ^a^P < 0.05; ^b^P < 0.01; ^c^P < 0.001.

To verify the expression pattern revealed by the RNA-seq analysis, qPCR was performed on the 27 down-regulated DEGs except DEG 9 (*AtOXS2*) with the same tissues used for RNA-seq. The expression pattern obtained by qPCR was not very consistent with the RNA-seq data, as the expression level of fourteen DEGs among the 27 down-regulated DEGs identified by RNA-seq did not decrease or decrease significantly (less than 2 fold). However, nearly half of the genes (13 DEGs) among the selected DEGs showed a dramatic change in gene expression (2.2- to 10.5-fold, Table 1). To explore the expression of the selected DEG expression in a greater detail, the expression level of the DEGs under the normal growth condition should also be detected. The mutants and wild-type plants were germinated on ½ MS media for 3 d, and then transferred to plates without or with 150 mM NaCl for another 10 d for RT-qPCR analysis of these thirteen dramatically decreased genes. As shown in Fig. 5, a number of salt-response genes were discovered. Some genes were over-expressed by salt stress in the wild-type plants, including DEG3, DEG6, DEG13, DEG18 and DEG19 (Fig. 5(b,e,h,i,j)). Among these genes, DEG6, DEG13 and DEG19 were not induced by salt stress in the absence of OXS2 (Fig. 5(b,h,j)), and the transcript abundance of DEG3 and DEG18 significantly decreased compared with that under the normal growth condition (Fig. 5(e,i)). It is suggested that OXS2 is the key upstream regulator of these five DEGs in response to salt stress. Besides the OXS2 related salt activated genes, some genes were down-regulated by salt stress in the wild-type plants, including DEG4, DEG10, DEG12, DEG24 and DEG28 (Fig. 5(c,f,g,k,m)). Four of these genes (DEG10, DEG12, DEG24 and DEG28) were expressed at a similar level without or with OXS2 under the normal growth condition, and DEG4 was significantly down-regulated in *oxs2-1*. However, all of these genes were dramatically down-regulated in the *OXS2* mutants under salt stress (Fig. 5(c,f,g,k,m)). It is indicated that OXS2 also played an essential role as an upstream regulator in response to salt stress; without OXS2, the genes were more sensitive to salt stress. A number of genes (DEG2, DEG5 and DEG25) did not show a significant difference of the expression levels upon salt stress in the wild-type plants (Fig. 5(a,d,l)). However, in the *OXS2* mutants, the expression of all of the DEGs was dramatically decreased (Fig. 5(a,d,l)). These results showed that, even in the absence of salt, all of these three genes were regulated by OXS2.

**Table 1:** Summary of down-regulated differentially expressed genes in WT VS *oxs2-1* comparison group discovered by RNA-seq. DEGs were ranked from top to bottom according to the fold change (WT VS oxs2-1) identified by qPCR. BOXS2 is a CT-rich motif in the putative promoter (2 Kb upstream of the coding region), which was calculated by a position frequency matrix tool according to the SAAB result^24^.

DEG Number	GeneID	Fold Change (qPCR) WT VS oxs2-1	BOXS2
DEG2	AT3G44006	10.466369	CTTCTTCTC (1386-1394)
DEG3	AT4G29200	10.464263	CTTCTTCTC (1143-1151)
DEG4	AT4G34790	9.5341629	CTTCCTTTC (892–900)
DEG18	AT4G12980	6.902787	CTTCTTTTC (1609–1617)
DEG10	AT3G01500	5.0696121	CTCGCTCTC (1427–1435) CTTCTTCTC (1525–1533) CTTCTTTTC (1927–1935)
DEG19	AT5G25980	3.6420667	
DEG24	AT5G38420	3.619712	CTTCTTCTC (1089–1097)
DEG5	AT3G05945	3.2440316	
DEG6	AT3G30720	3.0215489	CTCCTTTTC (1951–1959)
DEG13	AT3G27690	2.918036	CTCGCTCTC (1022–1030)
DEG12	AT5G14740	2.7840692	CTCCCTTTC (931–939)
DEG25	AT1G29395	2.4340653	
DEG28	AT3G05727	2.1557582	CTTCTTCTC (1199–1207)
DEG16	AT4G14400	1.8827915	
DEG7	AT2G42380	1.8514639	CTTCTTCTC (153–161)
DEG1	AT3G49230	1.5516772	
DEG23	AT1G11120	1.1261533	CTTCCTCTC (1938–1946)
DEG22	AT4G26530	1.0748932	
DEG15	AT5G48490	1.044392	
DEG20	AT4G02850	1.0277407	CTCCTTGTC (1589–1597)
DEG11	AT2G34430	0.9983498	
DEG14	AT2G29290	0.9854896	
DEG21	AT2G34170	0.9825152	
DEG8	AT5G38930	0.9305307	
DEG27	AT1G62500	0.8855457	
DEG26	AT1G73600	0.8696551	
DEG17	AT1G10657	0.8609541	CTTCTTTTC (174–182)
DEG9	AT2G41900		CTCCCTCTC (375–383) CTCCTTCTC (476–483)

Numbers in the parenthesis represent the position of the motif from 2 Kb before the coding region.

### Salinity related pathways identified from RNA-Seq analysis

Salt-stress responses are always related to photosynthesis. Strong photosynthesis can enhance salt tolerance in some plant species. For example, increased photosynthesis confers salt stress tolerance in watermelon^26^. In our transcriptomic analysis, three of the 13 down-regulated DEGs are related to photosynthesis (Table 2). Salt stress also interrupts respiration and carbon metabolism in plants; efficient respiration is imperative for salinity tolerance^15^. Our analyses show that four of the 13 down-regulated DEGs are involved in carbon metabolism (Table 2). These results indicated that AtOXS2 might play a role in salt resistance through regulating photosynthesis and carbon metabolism. Salinity stress can induce oxidative damage in many plant species. Oxidative elements, such as ROS, play an important role in oxidative homeostasis and signaling pathways in response to salt stress^27^. As shown in Table 2, two of the 13 DEGs are involved in the oxidation-reduction process. ABA-signaling is the central regulation pathway of salt-stress responses; salt stress treatment can be activated by ABA responses in plants^28,29^. In our results, two of the 13 down-regulated DEGs are involved in responses to ABA (Table 2). It is suggested that oxidative stress and ABA response related genes may also be regulated by AtOXS2.

**Table 2:** Summary of down-regulated differentially expressed genes in WT VS *oxs2-1* comparison group identified by qPCR (less than 2 fold).

DEG Number	GeneID	Log_2_ Ratio WT VS *oxs2-1*	Fold Change WT VS *oxs2-1*		Results of blast against nr database	TAIR Annotation (Key Words)
DEG2	AT3G44006	−5.2148	0.026927463		hypothetical protein	This gene encodes a small protein and has either evidence of transcription or purifying selection.
DEG3	AT4G29200	−4.8655	0.034304627		Beta-galactosidase related protein	Beta-galactosidase related protein
DEG4	AT4G34790	−3.6286	0.080848939		SAUR-like auxin-responsive protein family	response to auxin
DEG5	AT3G05945	−2.5701	0.168388441			
DEG6	AT3G30720	−1.5808	0.334298315		qua-quine starch	negative regulation of starch metabolic process, positive regulation of protein metabolic process, starch biosynthetic process
DEG10	AT3G01500	−1.3002	0.406068198	**α**, **β**	carbonic anhydrase 1	carbon utilization, defense response to bacterium, defense response to fungus, incompatible interaction, negative regulation of stomatal complex development, photosynthesis, regulation of stomatal movement, response to carbon dioxide, response to cold
DEG12	AT5G14740	−1.1985	0.435715601	**β**	carbonic anhydrase 2	carbon utilization, defense response to bacterium, regulation of stomatal movement, response to carbon dioxide
DEG13	AT3G27690	−1.1934	0.437262295	**α**	photosystem II light harvesting complex protein 2.3	response to blue light, response to cold, response to desiccation, response to far red light, response to high light intensity, response to light stimulus, response to low light intensity stimulus, response to red light
DEG18	AT4G12980	−1.1046	0.465026703	**χ**	Auxin-responsive family protein	multicellular organism development, oxidation-reduction process
DEG19	AT5G25980	−1.0972	0.467436341	**β**, **δ**	glucoside glucohydrolase 2	abscisic acid-activated signaling pathway, carbohydrate metabolic process, defense response to insect, glucosinolate catabolic process, regulation of stomatal movement, response to abscisic acid, response to salt stress
DEG24	AT5G38420	−1.0376	0.487144309	**α**, **β**, **χ**	Ribulose bisphosphate carboxylase (small chain) family protein	carbon fixation, oxidation-reduction process, photorespiration, reductive pentose-phosphate cycle, response to blue light, response to far red light, response to red light
DEG25	AT1G29395	−1.0314	0.489218757	**δ**	COLD REGULATED 314 INNER MEMBRANE 1	cellular response to cold, cellular response to water deprivation, cold acclimation, response to abscisic acid
DEG28	AT3G05727	−1.0069	0.497612635		S locus-related glycoprotein 1 (SLR1) binding pollen coat protein family	defense response to fungus, killing of cells of other organism

**α**: description of photosynthesis, **β**: description of carbon metabolism, **χ**: description of oxidation-reduction process, **δ**: description of response to ABA.

### Most significantly down-regulated DEGs are driven by *cis*-elements containing BOXS2 motifs

As a transcription factor, OXS2 mainly binds the DNA sequence containing a 9-bp CT rich motif, namely BOXS2, *in vitro*^24^. To predict the binding condition between OXS2 and the 27 selected genes *in vivo*, a computational framework for transcription factor binding site analysis (TFBS) was conducted using the *Arabidopsis* genome (Supplementary Table S2). As shown in Table 1, most genes with a dramatically decreased expression upon salt stress contained a BOXS2 motif except DEG5, DEG19 and DEG25. The DEGs with the most dramatic decrease of expression contained more BOXS2 motifs compared with the other genes. These results suggested that OXS2 regulated the downstream DEGs mainly through binding the BOXS2 motif. DEG5, DEG19 and DEG25, which do not contain a BOXS2 motif, were probably affected by other DEGs which were directly regulated by OXS2. Some of the putative OXS2-binding DEGs were constitutively activated by OXS2, including DEG2, DEG3, DEG4, and DEG6 (Fig. 5(a–c,e)). The others were activated by OXS2 under the treatment of salt, including DEG10, DEG12, DEG13, DEG18, DEG24 and DEG28 (Fig. 5(i,k,m)). Out of the three DEGs without BOXS2, DEG19 and DEG25 were involved in response to salt tolerance. Generally, at the molecular level, there are mainly two salt tolerance regulation manners. One is that the transcription factor constitutively binds the downstream salt tolerant DEGs without or with salt stress. The other is that, when the plant is treated with salt stress, the transcription factor expressed in the cytoplasm specifically enters the nuclear and activates other downstream salt tolerant DEGs.

### AtOXS2 associates with *CA1* and *Araport11* directly

**Figure 6:**
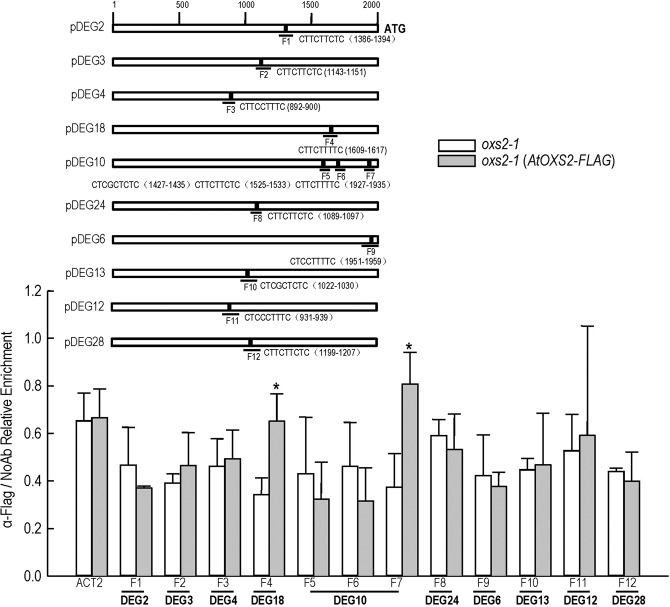
ChIP-qPCR to test *in vivo* interaction of promoters (including 5’UTR) with AtOXS2 in 10-d-old seedlings from *oxs2-1* and *oxs2-1* (AtOXS2-FLAG) treated with 150 mM NaCl. Promoter or segments tested are labeled F1-F12. CP (crossing point) value of immuno-precipitated DNA fractions with α-FLAG or no antibody control (NoAb) normalized to CP value of input DNA fractions for the same qPCR assay. Y axis is the ChIP signals calculated as the enrichment relative to the no-antibody control (No Ab). Error bars indicate ± SD from three independent experiments. P value of Student’s t test: *oxs2-1* (AtOXS2-FLAG) compared with *oxs2-1*. *P < 0.05; ***P < 0.001.

To explore the interaction between the BOXS2-containing promoters and AtOXS2, we generated transgenic *Arabidopsis oxs2-1* producing an FLAG-tagged AtOXS2, which can recover the salt sensitive phenotype (Supplementary Fig. S2). After identifying the expression of the fusion protein by western blot (Supplementary Fig. S3), a chromatin immunoprecipitation-quantitative PCR (ChIP-qPCR) analysis was performed to test the *in vivo* interaction of these promoters in *oxs2-1* and *oxs2-1* (AtOXS2-FLAG). Following immunoprecipitation with anti-FLAG antibody, twelve pairs of primers were used for the ten promoters corresponding to fragments F1-F12 (Fig. 6). Positive interaction with AtOXS2 was found for F4 and F7 (Fig. 6), but not for the other fragments, including the *ACT2* (At3g18780) promoter used as the negative control. It is indicated that AtOXS2 could bind the promoters of DEG10 (*carbonic anhydrase 1*, *CA1*) and DEG18 (*Auxin-responsive family protein gene*, *Araport11*) directly. *CA1* is induced by salt stress in *Dunaliella salina*, and the promoter of *CA1* is able to drive a stable expression of two foreign genes (*BAR* and *GUS*) in transformed cells of *D*. *salina* under salt stress^30^. Araport11 (NAC domain containing protein 1) is a member of the NAC transcription factor family. *Araport11* of *Suaeda liaotungensis* K (*SlNAC1*) can enhance salt tolerance in *Arabidopsis*^31^. The other fragments of promoters were possibly regulated through the *cis*-elements of AtOXS2 or unknown interactive proteins of the transcript factors.

## Conclusion

**Figure 7:**
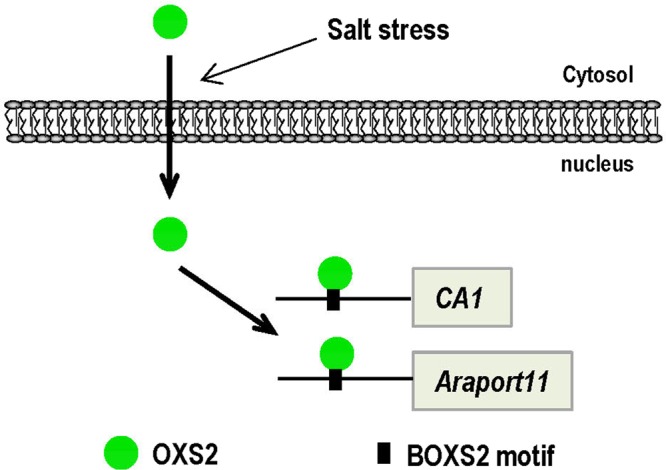
Model of OXS2 regulation of salt stress.

Although OXS2 was proved to be responsive to several types of stresses, such as ABA, cold, diamide and Cd^24,25^, there is no report indicating that OXS2 was involved in salt stress. In this paper, we found the loss of *OXS2* led to a salt-sensitive phenotype of *Arabidopsis* with multiple disrupted pathways or molecular functions. Further analysis identified that OXS2 regulated salt tolerance mainly through associating with BOXS2 motif containing DEGs, such as *CA1* and *Araport11*, which are related to salt responses. We found that both of DEG10 and DEG18 were down regulated in *oxs2-1* under salt stress (Fig. 5(f,i)). These DEGs are different from the constitutively down-regulated DEGs, such as DEG2, DEG3, DEG4, DEG5 and DEG6 (Fig. 5(a–e)), which showed a decreased level in *oxs2-1* under the normal growth condition. It is consistent with the protein localization of OXS2 without or with salt stress (Fig. 3). These results suggest that the salt tolerance regulated by OXS2 was specifically induced by salt stress in *Arabidopsis*. Without stress, OXS2 localized in cytoplasmic; when treated with salt, OXS2 entered the nuclear and associated with the promoters of *CA1* and *Araport11*, and played a role in salt tolerance in *Arabidopsis* (Fig. 7).

## Methods

### Plant culture and treatment

*Arabidopsis thaliana*, wild-type Col-0 (SALK_6000) and T-DNA insertion mutant *oxs2-1* (SALK_037470) have been described previously^24^. *Arabidopsis* plants were grown in a controlled environment at 22 °C/20 °C in a 16-h-light/8-h-dark photoperiod. Seeds used for phenotypic assays were harvested at the same time. The tolerance test was performed on plates with ½ MS solid media without or with 150 mM NaCl. The cultured seeds were germinated in plates without stress for 3 d, and then transferred to plates with 150 mM NaCl for another 10 d.

For the salt-inducible experiment, the *Arabidopsis* plants were germinated and grown in ½ MS. Ten-day-old seedlings were transferred to ½ MS hydroponic cultures with 150 mM NaCl, and 10 seedlings were collected at different time points from the start of the salt treatment.

### Seed germination bioassay

*Arabidopsis* seeds were planted in ½ MS and placed at 4 °C for 3 d. Emerge-germinating seeds were counted in a controlled environment at 22 °C/20 °C in a 16-h-light/8-h-dark photoperiod at different time points.

### Molecular constructs

For the transient expression construct, the *AtOXS2* coding region without the stop codon was PCR amplified from *Arabidopsis* (Col) cDNA with the primer pair of AtOXS2 F: 5′ GCGTCGACATGTGCTGTGGATCAGACC 3′ and AtOXS2 R: 5′ CGGGATCCTCAATTCTGCTGAGCCACA 3′. The amplified fragment was inserted into the transient expression vector pGFP using the *SacI* and *BamHI* sites. For the expression construct of AtOXS2, the coding region was PCR amplified from *Arabidopsis* (Col) cDNA with the primer sets AtOXS2-1F/AtOXS2-1R. The DNA was inserted into the *XbaΙ* site of the binary vector pCambia3300 (http://www.cambia.org) to yield p35S::AtOXS2 using the In-Fusion HD cloning kit (catalog No. 011614; Clontech). To make the 35S::AtOXS2-FLAG construct, the AtOXS2 coding region without its stop codon was PCR amplified from genomic DNA with the primers AtOXS2-2F/AtOXS2-R and inserted between the *KpnI* and *PstI* sites on pCambia1305. All primers are listed in Supplementary Table S3.

### Protein subcellular localization assay

Transient expression in onion epidermal cells was carried out using the Biolistic PDS 1000/He Particle Delivery System (Bio-Rad) (1100 psi, 10 cm traveling distance) with plasmid DNA, 0.5 M CaCl_2_ and 10 mM spermidine precipitated onto 1 mm gold particles. Plasmids were delivered onto onion epidermis cultured by ½ MS without or with 150 mM NaCl. After incubating for about 10 h in the dark at 25 °C, the epidermis was observed under a Zeiss 300 confocal microscope.

### RT-qPCR

RNA extraction was conducted using a plant RNA kit (catalog No. R5105; GBCBIO Technologies). Reverse transcription was conducted using PrimeScript RT reagent kit with a gDNA Eraser (catalog No. RR047A; TaKaRa). qPCR was conducted with SYBR Premix Ex Taq (catalog No. DRR820A; TaKaRa) on a LightCycler 480 II (Roche). *Arabidopsis ACT2* (*AT3G18780*) was used as an internal control, and the 2^−ΔΔCT^ method was used in the analysis of the real time PCR data. All primers are listed in Supplementary Table S3.

### RNA-Seq library construction and sequencing

The wild-type plants and *oxs2-1* were germinated on ½ MS plates for 3 d, and then transferred to plates with 150 mM NaCl for another 10 d. Three independent batches of the 10-d-old plantlets were collected, and then stored in −80 °C before sending to Novogene for RNA-seq analysis. Total RNA isolation, library construction, sequencing, and basic data analysis were carried out by Novogene.

### Western blot

*Arabidopsis* leaves were harvested and ground with 2 mL of immunoprecipitation buffer (50 mM TrisHCl, pH 7.5, 150 mM NaCl, and 1% [v/v] Triton X-100) with freshly prepared DTT (1 mM) and 1X protease inhibitor cocktail (catalog No. 04693132001; Roche) on ice. Protein extracts were centrifuged at 12,000 g for 30 min at 4 °C. A total of 100 μL of supernatant was stored at −80 °C for immunoblot. Protein samples (20 μL) were loaded onto 10% (w/v) SDS-PAGE gels and the immunoblot was conducted as described before^32^.

### Chromatin immunoprecipitation

*Arabidopsis* seeds were germinated in ½ MS for 3 d, and then the same size seedlings were transferred to the ½ MS with 150 mM NaCl and cultured for another 7 d. About one gram fresh tissue (whole seedlings) was collected to perform chromatin immunoprecipitation as described before^25^. All primers are listed in Supplementary Table S3.

### Accession numbers

Sequence data for the RNA-seq samples can be found in the NCBI’s Sequence Read Archive (SRA) database under the following accession number: SUB4904863.

## Supplementary information

Supplementary Information.

Supplementary Information2.
