# Supplementary material for: OXS2 is Required for Salt Tolerance Mainly through Associating with Salt Inducible Genes, CA1 and Araport11, in Arabidopsis
Source: Sci Rep. 2019 Dec 30;9:20341. doi: 10.1038/s41598-019-56456-1 (PMC6937310; doi:10.1038/s41598-019-56456-1)
Supplement: Supplementary file 2 — Supplementary Information2. [file 41598_2019_56456_MOESM2_ESM.pdf]

OXS2 is Required for Salt Tolerance Mainly through Associating with Salt Inducible Genes: *CA1* and *Araport11* in *Arabidopsis*

Ying Jing[1,2,3] , Lin Shi[1,3], Xin Li[1], Han Zheng[1], Jianwei Gao[1,2], Mei Wang[1,2], Lilong He[1,2*] and Wei Zhang[1,2*]

[1] Institute of Vegetables and Flowers, Shandong Key Laboratory of Greenhouse Vegetable Biology, Shandong Branch of National Vegetable Improvement Center, Huanghuai Region Vegetable Scientific Station of Ministry of Agriculture (Shandong), Shandong Academy of Agricultural Sciences, Jinan 250100, China

[2] Key Laboratory of Plant Development and Environment Adaptation Biology, Ministry of Education; School of Life Science, Shandong University, Qingdao, 266237, China

[3] These authors contributed equally to this work.

[*] Correspondence: hllong1984@163.com (L.H.); weizhang@sdu.edu.cn (W. Z.)

Tel.: +86-531-6665-9060 (L. H.); +86-13188933448 (W. Z.)

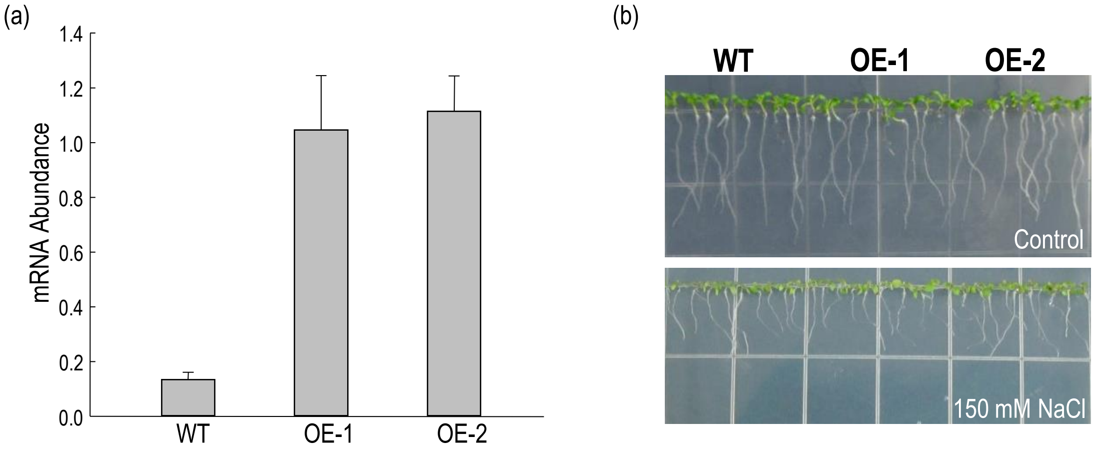

Supplementary Figure 1. Phenotyping analysis of *OXS2* overexpressing (OE) lines and wild-type plants. (a) Transcript abundance identification. Expression of *OXS2* (relative to ACT1 control) in the leaves of 4-w-old wild-type and overexpressing lines. Error bars indicate ± SD of three independent experiments（three leaves from one plant). (b) Arabidopsis plants germinated on ½ MS plates vertically for 3 d were transferred to plates without or with 150 mM NaCl for another 10 d.

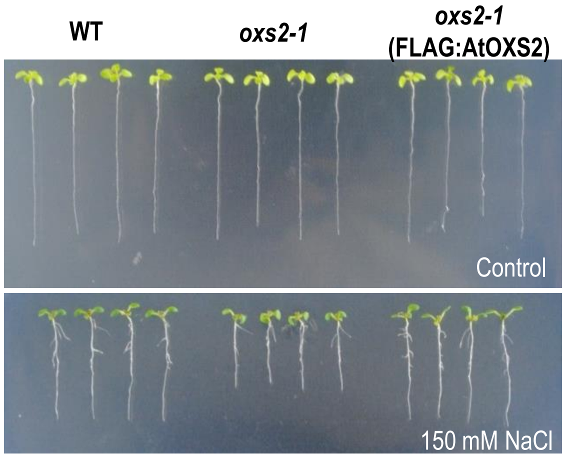

Supplementary Figure 2: Phenotyping analysis of *oxs2-1*, and wild-type plants without or with salt stress. Arabidopsis plants germinated on ½ MS plates vertically for 3 d were transferred to plates without or with 150 mM NaCl for another 10 d. Representative result from three reproducible experiments was shown.

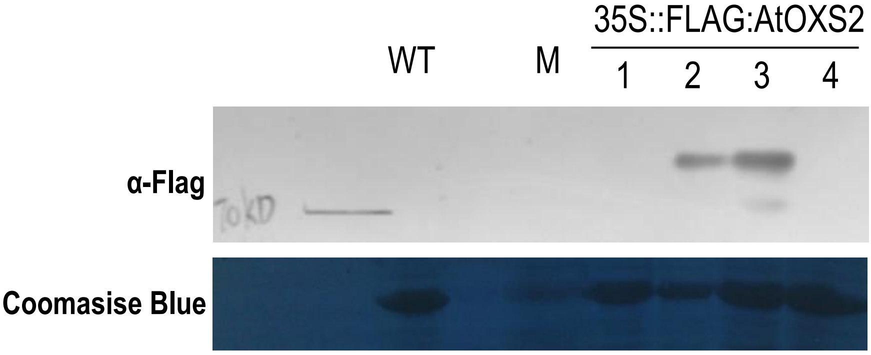

Supplementary Figure 3. Western blot of *oxs2-1* expressing FLAG:AtOXS2 lines and wild type plants. Total protein extracted from 4-week-old leaves of wild-type plant and independent transgenic plants which identified by genomic PCR. The anti-FLAG antibody which described before[25] was used to detect FLAG::AtOXS2. Total protein stained by coomasise blue was used as control. The blots were cut from the same images separately. Representative results from three reproducible experiments are shown. WT, Wild type.

Supplementary Table S1. Down-regulated differentially expressed genes in WT VS oxs2-1 comparison group discovered by RNA-seq

DEG Number	GeneID	Log_2Ratio (WT VS oxs2-1)	Fold Change WT VS oxs2-1
**DEG1**	AT3G49230	-5.7398	0.018712572
**DEG2**	AT3G44006	-5.2148	0.026927463
**DEG3**	AT4G29200	-4.8655	0.034304627
**DEG4**	AT4G34790	-3.6286	0.080848939
**DEG5**	AT3G05945	-2.5701	0.168388441
**DEG6**	AT3G30720	-1.5808	0.334298315
**DEG7**	AT2G42380	-1.373	0.386080525
**DEG8**	AT5G38930	-1.3383	0.395480201
**DEG9（AtOXS2）**	AT2G41900	-1.3144	0.402094314
**DEG10**	AT3G01500	-1.3002	0.406068198
**DEG11**	AT2G34430	-1.2206	0.429100856
**DEG12**	AT5G14740	-1.1985	0.435715601
**DEG13**	AT3G27690	-1.1934	0.437262295
**DEG14**	AT2G29290	-1.1781	0.441918368
**DEG15**	AT5G48490	-1.1652	0.445907935
**DEG16**	AT4G14400	-1.1086	0.463747396
**DEG17**	AT1G10657	-1.1065	0.464432893
**DEG18**	AT4G12980	-1.1046	0.465026703
**DEG19**	AT5G25980	-1.0972	0.467436341
**DEG20**	AT4G02850	-1.0783	0.473578589
**DEG21**	AT2G34170	-1.0651	0.477941415
**DEG22**	AT4G26530	-1.0584	0.480156926
**DEG23**	AT1G11120	-1.0418	0.485716427
**DEG24**	AT5G38420	-1.0376	0.487144309
**DEG25**	AT1G29395	-1.0314	0.489218757
**DEG26**	AT1G73600	-1.0281	0.490368845
**DEG27**	AT1G62500	-1.0109	0.49623995
**DEG28**	AT3G05727	-1.0069	0.497612635

Supplementary Table S3. Primer Sequences. Note: lowercase means sequences from vectors and capital letter means sequences from genes.

primer name	Sequence	primer name	Sequence
**AtOXS2 -1F**	5' gtacccggggatcctGCGTCGACATGTGCTGTGGATCAGACC 3'	**DEG21 q R**	5'-GGCTGTCGCTTCCAAGAATA-3'
**AtOXS2 -1R**	5' gcaggtcgactctagCGGGATCCTCAATTCTGCTGAGCCACA 3'	**DEG22 q F**	5'-GAAGCCATGGACTCTCACTTT-3'
**AtOXS2 -2F**	5' GGGGTACCATGTGCTGTGGATCAGACCG 3'	**DEG22 q R**	5'-CCTGAGCTTTGGCTACATTCT-3'
**AtOXS2 -2R**	5' AACTGCAGATTCTGCTGAGCCACAAGCTGATC 3'	**DEG23 q F**	5'-GCAACGGCTATTCTCATCTCT-3'
**qRT-PCR analysis**		**DEG23 q R**	5'-CCCGACTTGAAGAGGAATCTG-3'
**DEG1 q F**	5'-ACGGTCACTTGGTTCATCAC-3'	**DEG24 q F**	5'-CAAGCAACGGAGGAAGAGTTAG-3'
**DEG1 q R**	5'-TTGGTTGTATCTCCTTCCCTTTC-3'	**DEG24 q R**	5'-GTCACTAAGGTCAGGGAGGTAA-3'
**DEG2 q F**	5'-ATGCTGGCACTTCACCAA-3'	**DEG25 q F**	5'-GCCTGGATTAAGGGAGAGTATG-3'
**DEG2 q R**	5'-CTATCCACCGTTTCAGGACTAAC-3'	**DEG25 q R**	5'-GCCACAATCACCAAGAGTAATG-3'
**DEG3 q F**	5'-CTGGATCTTTGCCACCTACTT-3'	**DEG26 q F**	5'-TCTCCAACTGGCTGCTAATG-3'
**DEG3 q R**	5'-CCAAGAAAGGCGAGATGATTTG-3'	**DEG26 q R**	5'-GGAAACAAGACTCCCGAAAGA-3'
**DEG4 q F**	5'-AAGGTCACGTAGCGGTTTAC-3'	**DEG27 q F**	5'-GTTAGACCTCCTCCCGTAGT-3'
**DEG4 q R**	5'-GCCACCCATAGAGTGATGAAA-3'	**DEG27 q R**	5'-TGATGGGAGGTCTTGTGATTG-3'
**DEG5 q F**	5'-AGCTCATAGACACATGTCTTAACAA-3'	**DEG28 q F**	5'-CCATCTGCTTCACCACTCTT-3'
**DEG5 q R**	5'-CCCGACGACAATGGAGTTAAT-3'	**DEG28 q R**	5'-TTTCCTTACATGGGTACACTGG-3'
**DEG6 q F**	5'-GTTGAAAGAAGCTTCAAACCAAAC-3'	**Actin2 qF**	5'-GGTAACATTGTGCTCAGTGGTGG-3'
**DEG6 q R**	5'-GACCCATGATATGACCCTCATT-3'	**Actin2 qR**	5'-AACGACCTTAATCTTCATGCTGC-3'
**DEG7 q F**	5'-CAGAGATCAAGGGTGAGGAAAC-3'	**ChIP-qPCR analysis**	
**DEG7 q R**	5'-GAATGCAACTCTTGGCGATAAC-3'	**actin2 F**	5' GTTAGCAACTGGGATGATATGG 3'
**DEG8 q F**	5'-TTGTCGAACTAGGAACACTTCTC-3'	**actin2 R**	5' CAGCACCAATCGTGATGACTTGCCC 3'
**DEG8 q R**	5'-GAGTCCCTCTGGAAACACAAA-3'	**DEG2 F1 F**	5' ATACAATTCATTTTTTCTCTTCTTT 3'
**DEG10 q F**	5'-TTCCGTTCCAACGCTTATCC-3'	**DEG2 F1 R**	5' ACTATATAAGGTTAGGCATGGCT 3'
**DEG10 q R**	5'-GTATGCTTCGGTTCCCATCTC-3'	**DEG3 F2 F**	5' TTGCTCTGTTTCTTCTTCTCT 3'
**DEG11 q F**	5'-CATAGCCAACCTTCCGTTCT-3'	**DEG3 F2 R**	5' ATTGACTCTTTTTCTCTATCTCTCT 3'
**DEG11 q R**	5'-GATGCCTTCGTGGTTCGTAATA-3'	**DEG4 F3 F**	5' AGATATTTGTGGAGAATTTTTGC 3'
**DEG12 q F**	5'-GATGCCTTCGTGGTTCGTAATA-3'	**DEG4 F3 R**	5' ACATTATATTGTTTCTGTTGAGTGA 3'
**DEG12 q R**	5'-AGGTGCAAGACAGCGTATTC-3'	**DEG18 F4 F**	5' TATCTAAAAACATGCATCTACACTA 3'
**DEG13 q F**	5'-CACACGTACTAGACTTCCATCC-3'	**DEG18 F4 R**	5' TACAATAAAATCTAAATTCACAACA 3'
**DEG13 q R**	5'-GGCTCCAACTCCTGCATATT-3'	**DEG10 F5 F**	5' TGCGTTACTGGTCTCTGTATTTCTT 3'
**DEG14 q F**	5'-CGTGAGCAACGAGAGAAACT-3'	**DEG10 F5 R**	5' CTCAGACCTTCCATGGCCATA 3'
**DEG14 q R**	5'-GTGGTCGGCTTGAGCATTA-3'	**DEG10 F6 F**	5' TGATGATTTATGGCCATGGAA 3'
**DEG15 q F**	5'-GGCTAGTTTAGTCGTTGAGAGG-3'	**DEG10 F6 R**	5' ATCGATCATGGACTGTTCTACTCTA 3'
**DEG15 q R**	5'-CTCGTCGGGTTATTCTTGCT-3'	**DEG10 F7 F**	5' CTTTCTGAGACCTTTTATTTTTTTA 3'
**DEG16 q F**	5'-TGAAGAGAGTGAGGGACTGAA-3'	**DEG10 F7 R**	5' ATAGCTGCGGTGAATAACAAAC 3'
**DEG16 q R**	5'-GACAGGTAGCCATCTCCAAATAG-3'	**DEG24 F8 F**	5' ATTAGATAGTTTACGATACATTCAA 3'
**DEG17 q F**	5'-CACAACACAACAATCTCCCTTG-3'	**DEG24 F8 R**	5' CACGTAAAAGCTCATATATG 3'
**DEG17 q R**	5'-CGCGTCATACGGAGTAGAATG-3'	**DEG6 F9 F**	5' TCTTAAATAAATGTCCAAGCTTG 3'
**DEG18 q F**	5'-AAGGTTCCGGCGGATTTAG-3'	**DEG6 F9 R**	5' TTTTTTGGATCGAAAGAAGTGAATA 3'
**DEG18 q R**	5'-CGAGTGATCCCACTGAGTTAAG-3'	**DEG13 F10 F**	5' TTTAATTTATCCAATCATATACTTT 3'
**DEG19 q F**	5'-ATTCTTCCACCGAACCCTATATC-3'	**DEG13F10 R**	5' ATAATGGAGCTGATTGGGAT 3'
**DEG19 q R**	5'-CAATCTTCCCTCCTTGGTACTT-3'	**DEG12 F11 F**	5' GGGATCTTCGATTTCGAC 3'
**DEG20 q F**	5'-CTGGAGCCTCAAGATGAACAA-3'	**DEG12 F11 R**	5' GACTTTCTTCTCTTATCCACATCA 3'
**DEG20 q R**	5'-CAAGACTCTCTGCTTCTCCTTATC-3'	**DEG28 F12 F**	5' ATATGTTGTTAGAGAATCGA 3'
**DEG21 q F**	5'-GTGGTGTTGAGGCAATGAAAC-3'	**DEG28 F12 R**	5' AATTCATTTATTTTAGTTACCTTTG 3'
